# The Value of Cardiopulmonary Exercise Testing in Determining Severity in Patients with both Systolic Heart Failure and COPD

**DOI:** 10.1038/s41598-020-61199-5

**Published:** 2020-03-09

**Authors:** Cássia da Luz Goulart, Polliana Batista dos Santos, Flávia Rossi Caruso, Guilherme Peixoto Tinoco Arêas, Renan Shida Marinho, Patricia de Faria Camargo, Tiago da Silva Alexandre, Claudio R. Oliveira, Andréa Lúcia Gonçalves da Silva, Audrey Borghi-Silva, Renata Gonçalves Mendes, Meliza Goi Roscani

**Affiliations:** 1 Cardiopulmonary Physiotherapy Laboratory, Physiotherapy Department, Federal University of Sao Carlos, UFSCar, Rod Washington Luis, KM 235, Monjolinho, CEP: 13565-905, Sao Carlos, SP Brazil; 2 Department of Gerontology, Federal University of São Carlos, São Carlos, Brazil; 3 Department of Physical Education and Health, University of Santa Cruz of Sul, Rio Grande do Sul, Brazil

**Keywords:** Cardiology, Cardiovascular diseases, Heart development

## Abstract

Our aim was to identify optimal cardiopulmonary exercise testing (CPET) threshold values that distinguish disease severity progression in patients with co-existing systolic heart failure (HF) and chronic obstructive pulmonary disease (COPD), and to evaluate the impact of the cut-off determined on the prognosis of hospitalizations. We evaluated 40 patients (30 men and 10 woman) with HF and COPD through pulmonary function testing, doppler echocardiography and maximal incremental CPET on a cycle ergometer. Several significant CPET threshold values were identified in detecting a forced expiratory volume in 1 second (FEV_1_) < 1.6 L: 1) oxygen uptake efficiency slope (OUES) < 1.3; and 2) circulatory power (CP) < 2383 mmHg.mlO_2_.kg^−1^. CPET significant threshold values in identifying a left ventricular ejection fraction (LVEF) < 39% were: 1) OUES: < 1.3; 2) CP < 2116 mmHg.mlO_2_.kg^−1^.min^−1^ and minute ventilation/carbon dioxide production (*V̇*_E_/*V̇*CO_2_) slope>38. The 15 (38%) patients hospitalized during follow-up (8 ± 2 months). In the hospitalizations analysis, LVEF < 39% and FEV_1_ < 1.6, OUES < 1.3, CP < 2116 mmHg.mlO_2_.kg^−1^.min^−1^ and *V̇*_E_/*V̇*CO_2_ > 38 were a strong risk predictor for hospitalization (P ≤ 0.050). The CPET response effectively identified worsening disease severity in patients with a HF-COPD phenotype. LVEF, FEV_1,_ CP, OUES, and the *V̇*_E_/*V̇*CO_2_ slope may be particularly useful in the clinical assessment and strong risk predictor for hospitalization.

## Introduction

Chronic obstructive pulmonary disease (COPD) and heart failure (HF) coexist not because of their high individual prevalence, but because both share common etiological and pathophysiological factors, such as smoking and systemic inflammation^1–3^. COPD overlap syndrome in HF can reach up to 30%^4^. The prevalence of COPD in patients hospitalized for HF is 10%, and the risk of developing HF during hospitalization due to COPD decompensation is 4.5%^5^. These diseases also have relevant systemic components that affect widely the musculoskeletal system^6–8^ in addition to the heart and lungs.

There is an increasing recognition that exercise intolerance in overlap of HF-COPD cases may be associated with increased ventilatory responses due to metabolic demand, resulting in ventilatory inefficiency^9,10^. This varies considerably in patients with HF-COPD with pulmonary involvement [Forced Expiratory Volume in the first second (FEV_1_)] and cardiac [left ventricular ejection fraction (LVEF)]^11–13^. However, the structural and physiological determinants that support this great variability remain poorly understood. It is not clear the impact of FEV_1_ and LVEF on cardiorespiratory and metabolic variables within cardiopulmonary exercise testing (CPET) in HF-COPD patients.

CPET is the gold standard and established tool to assess functional capacity and to determine prognosis in HF and COPD patients^14^. In addition, variables such as peak oxygen uptake (*V̇*O_2_), the product of peak ventilation (*V̇*_E_) and carbon dioxide production (*V̇*_E_/*V̇*CO_2_ slope)^14,15^ are important cardiac prognostic indices^13^. Guazzi *et al*. (2013) showed that the peak of *V̇*O_2_ < 10 mL/kg/min and *V̇*_E_/*V̇*CO_2_ slope ≥45 were independent predictors of long-term mortality in HF and COPD^14,15^. O_2_ pulse, circulatory and ventilatory power (CP and VP, respectively) are related to predictors of mortality in all chronic heart failure patients^16–18^.

In addition, the studies that performed CPET focused on the variation between different populations presented as main objective the comparison with tests^10–12,19,20^. Thus, there is no information about the impact of HF-COPD on CPET variables, since these patients have marked ventilatory and cardiac limitations and, from a clinical point of view, established cut-off values for these patients based on the disease severity. It would be of utmost importance to determine during the performance of CPET, the clinical condition, diagnosis and best prescription of cut-off training for these patients.

Our aim was to identify optimal CPET threshold values that distinguish disease severity progression in patients with co-existing systolic HF and COPD. Our secondary aim was to evaluate the impact of the cut-off determined on the prognosis of hospitalizations of these patients. We hypothesize that CPET variables are important prognostic determinants for cardiorespiratory worsening in patients with FEV_1_ < 1.6 and LVEF < 39%.

## Methods

### Study design

This cross-sectional study was designed following the recommendations of the STROBE statement. All patients were recruited from the Cardiology and Pulmonology outpatient clinic of São Carlos. The study followed the Declaration of Helsinki and was approved by the local ethics committees (Federal University of Sao Carlos) (protocol number: 91088318.7.1001.5504). All volunteers signed a written informed consent statement prior to participation.

### Subjects

51 patients with clinical diagnosis of COPD by pulmonary function test [FEV_1_/forced vital capacity (FVC) ratio of 0.7; FEV_1_ 60% of predicted] without previous COPD exacerbation (3 months before the study) and clinical diagnosis of HF from a cardiologist with ejection fraction (<50% by echocardiogram), without cognitive impairment or comprehension deficiencies, older than 50 years of age and with HF class I, II or III according to the New York Heart Association Functional Classification (NYHA) were included in the study^16^.

Some HF-COPD patients were excluded from this study, as follows: patients with musculoskeletal disorders or neurological conditions affecting the locomotor system in a way that precluded them from protocol participation, patients recently hospitalized with clinical diagnoses of lung cancer, heavy alcohol drinkers, any patient with observed complex cardiac arrhythmias or electrocardiogram alterations, and patients with uncontrolled metabolic diseases, such as diabetes mellitus.

According to GOLD (2016)^1^ the mean FEV_1_ value of COPD patients is 1.7 L. Thus, we emphasize that our results are based on the average of our study, which is similar to the recommended by GOLD for FEV_1_ 1.6 ± 0.1 L and LVEF < 39%.

### Protocol

All patients underwent an echocardiogram administered by a cardiologist, a pulmonary function exam performed by a pulmonologist, and a clinical assessment. Every patient completed the comprehensive evaluation process in two days: (1) clinical evaluation by a physician and a physical therapist, followed by lung function test and Doppler echocardiography; (2) CPET; (3) follow-up.

### Measurements

#### Doppler echocardiography

Initially for the clinical and diagnostic stratification, the HF-COPD patients were submitted to a 2D-echocardiogram using an iE33 system (Philips, Andover, MA, USA) with a 2–5 MHz matrix transducer and tissue Doppler imaging software. Quantification of the cardiac chambers was performed according to the American Society of Echocardiography. In our study we only included patients with reduced LVEF (<50%). Patients who presented preserved LVEF (>50%) or diastolic HF were excluded from study^16^.

#### Pulmonary function

The pulmonary function was assessed using a digital spirometer (Breeze®, Medgraphics, MGC Diagnostics Corporation, St. Paul, MN, EUA) that provided measures of the forced expiratory volume in the 1^st^ second (FEV_1_) and the forced vital capacity (FVC), enabling the calculation of the FEV_1_/FVC ratio. Spirometry was performed according to the recommendations of the American Thoracic Society/European Respiratory Society guidelines. The classification of severity of airflow limitation in COPD was assessed according to the Global Initiative for Chronic Obstructive Lung Disease (GOLD) recommendations, and patients were classified as moderate (GOLD II), severe (GOLD III), or very severe (GOLD IV)^1^.

#### Cardiopulmonary exercise testing

In accordance with the American College of Cardiology and American Heart Association Guidelines^15^, a physician and physical therapist supervised the CPETs and the subjects were asked to maintain routine medications on the day of testing. The test was performed on an electronically braked cycle ergometer (Corival Recumbent, Medical Graphics Corporation, St. Paul, Mo, USA) and respiratory gas analysis was measured breath-by-breath with Oxycon Mobile (Mijnhardt/Jäger, Würzburg, German).

The protocol consisted of the following: (I) 5-minute rest period while sitting on the cycle ergometer; (II) 1-minute exercise at free-wheel and 60 rotations per minute (rpm); (III) incremental phase with an increase of 5–10 W/min (ramp protocol); (IV) 1-minute of active recovery at free-wheel; and (V) 5-minute passive recovery resting in sitting position. A twelve-lead Electrocardiogram (ECG) was continuously monitored throughout the test (WinCardio, Micromed, Brasilia, Brazil). The test was finished when subjects were pedaling at their maximum possible effort level (physical exhaustion) and reported at least 2 of the following criteria: (I) age predicted maximal HR (220 j [age]); (II) general/leg fatigue or dyspnea; (III) angina or electrocardiographic evidence of ischemia or malignance arrhythmia (ventricular tachyarrhythmia, ventricular fibrillation, bigeminism); or (IV) inability to maintain a pedaling rate of 60 rpm for 30 seconds.

#### Ventilatory and hemodynamic measurements during CPET

During CPET the following parameters were measured: peak systolic and diastolic blood pressure (SBP and DBP) (mmHg), peak *V̇*O_2_ (ml/kg/min), *V̇*CO_2_ (ml/min), *V̇*_E_/*V̇*CO_2_ slope^21^, workload (WR) (watts), HR peak (bpm) and *V̇*O_2_ efficiency slope (OUES)^22^. O_2_ pulse was calculated using the product of peak *V̇*O_2_ and peak HR. CP was calculated using the product of peak *V̇*O_2_ and peak SBP^17^. VP was obtained by dividing peak SBP by the *V̇*_E_/*V̇*CO_2_ slope^21^. *V̇*O_2_/WR was determined by the relationship between maximal workload obtained and *V̇*O_2_ peak^23^.

#### Follow-up

Patients were included and accompanied during 12 months by telephone calls to their home or family physician. The hospitalizations were determined when the patient was hospitalized for more than one day, our patients had the following hospitalizations: COPD exacerbations (type II and III) (n = 6), HF decompensation (n = 5) and myocardial revascularization (n = 4). COPD exacerbations are classified as: type II - increased medication and medical intervention; and type III - worsening of the clinical condition requiring hospitalization^1^.

### Statistical analysis

The Shapiro-Wilk test was used to verify the data distribution. Descriptive data was shown as a mean, standard deviation and frequency. The parametric Student’s t-test was used for normally distributed data. Pearson correlation analysis were performed to investigate the relationship between variables. All tests were made in Statistical Package for the Social Sciences (SPSS) and values were accepted as P ≤ 0.05.

#### ROC curve

First, receiver operating characteristic (ROC) curve analyses selected the optimal threshold values to differentiate the severity of COPD considering FEV_1_ (L) and the severity of HF considering LVEF (%). Cut-off points discriminated the precision of CPET variables: VP, CP, O_2_ pulse, OUES, *V̇*_E_/*V̇*CO_2_ slope and *V̇*O_2_ peak in determining points of predictive cut-off in HF-COPD. The confidence interval (95% CI) was used to determine the ability of the clinical variables, with the lower limit being greater than 0.50. Subsequently, the cut-off points of the variables that obtained significant areas under the ROC curve were identified, with the respective values of sensitivity and specificity.

#### Kaplan-meier

We examined all hospitalization that occurred during the 12-months follow-up. Hospitalization curves were analyzed according to the Kaplan-Meier method to explore the impact of FEV_1_ < 1.6 and LVEF < 39%, CP < 2338, VE/VCO_2_>38 and OUES>1.3. Differences between curves were evaluated using the log-rank test.

## Results

### General characteristics

We initially included 51 HF-COPD patients, however due to our exclusion criteria, 11 patients did not participate in the study protocol: 1 was excluded for brain cancer, 7 did not agree to participate in the study, 1 due to aortic aneurysm, and 2 due to encephalic vascular accident. Therefore the protocol was carried out with 40 HF-COPD patients.

Table 1 presents the clinical, echocardiogram and spirometry’s characteristics in HF-COPD patients, 40 adult men and 10 woman with beta blocker and beta-agonists use (Table 1).

**Table 1:** Clinical, echocardiogram and spirometry’s characteristics in HF-COPD patients.

Variables	HF-COPD (N = 40)
Male, n (%)	30 (75)
Woman, n (%)	10 (25)
Age (years)	66 ± 8
Weight (kg)	71 ± 23
LVEF (%)	39 ± 8
**Medications, n (%)**	
β-Blocker	40 (100)
Β_2_-agonists	40 (100)
Diuretics	20 (50)
**Pulmonary Function**	
FEV_1_, L	1.6 ± 0.1
FVC, L	2.2 ± 1
FEV_1_/FVC, L	0.56 ± 0.1

Notes: *Mean ± SD; HF: heart failure; COPD: chronic obstructive pulmonary disease; LVEF: left ventricular ejection fraction; FVC: Forced Vital Capacity; FEV_1_, Forced Expiratory Volume in the 1 second; FEV_1_/FVC = Forced Expiratory Volume in the 1 second /Forced Vital Capacity.

During follow-up (10 ± 2 months), 15 patients were hospitalized (7 ± 2 months) and 25 were not hospitalized (10 ± 1 months). Table 2 expresses metabolic, ventilatory and hemodynamic variables of CPET in HF-COPD patients with hospitalizations [n = 15 (38%)] and non-hospitalizations [n = 40 (62%)]. We reported that the most hospitalized patients were those with FEV_1_ < 1.6 L [11 (71%)] and presented reduced O_2_ pulse when compared with non-hospitalized (p < 0.05).

**Table 2:** Metabolic, ventilatory and hemodynamic variables of CPET in HF-COPD patients.

Variables	HF-COPD (N v= 40)	Hospitalizations (N = 15)	Non Hospitalizations (N = 25)	P value
FEV_1_ (L)	1.6 ± 0.1	1.4 ± 0.6	1.7 ± 0.9	0.27
FEV_1_ < 1.6				0.05
Yes	21 (52)	11 (71)	10 (40)	
No	19 (48)	4 (29)	15 (60)	
LVEF < 39%				0.46
Yes	19 (48)	9 (60)	12 (48)	
No	21 (52)	6 (40)	13 (52)	
LVEF (%)	39 ± 8	36 ± 8	41 ± 9	0.53
WR peak (W)	60 ± 20	56 ± 16	63 ± 23	0.40
V̇_E_ peak (L/min)	39 ± 8	28 ± 9	38 ± 20	0.08
V̇O_2_ peak (ml.kg^−1^.min^−1^)	12 ± 3	11 ± 3	12 ± 3	0.51
V̇_E_/V̇CO_2_ slope	38 ± 10	40 ± 10	35 ± 11	0.22
O_2_ pulse (ml.bpm^−1^)	10 ± 5	8 ± 2	13 ± 8	0.01
VP (mmHg)	4 ± 1	4.9 ± 2	4.5 ± 1	0.20
CP (mmHg.mlO_2_.min^−1^)	2045 ± 727	1833 ± 708	2172 ± 722	0.15
OUES	1.3 ± 0.3	1.2 ± 0.4	1.3 ± 0.3	0.74
HR peak (bpm)	111 ± 24	114 ± 25	105 ± 20	0.24
SBP peak (mmHg)	165 ± 39	171 ± 40	162 ± 39	0.54
DBP peak (mmHg)	93 ± 26	105 ± 32	87 ± 19	0.03

Notes: *p < 0.05 = FEV_1_ > 1.9 vs FEV_1_ < 1.9; ^#^p < 0.05 = LVEF < 39% vs FEV_1_ < 1.9; ^†^p < 0.05 = LVEF < 39% vs LVEF > 39%; HF: heart failure; COPD: chronic obstructive pulmonary disease; HR: heart rate; WR: work rate; *V̇*O_2_: oxygen uptake; RER: respiratory exchange ratio; *V̇*_E_: Minute ventilation; *V̇*CO_2_: carbon dioxide production; *V̇*E/*V̇*CO_2_ slope: linear relation between minute ventilation and carbon dioxide production; OUES: Oxygen uptake efficiency slope; CP: circulatory power; VP: ventilatory power. HRR 1: Peak - Heart rate recovery in the first minute; SBP: Systolic blood pressure, DBP: Diastolic blood pressure.

We found significant correlations between: OUES *vs* LVEF (p = 0.018; r = 0.371) and FEV_1_ vs *V̇*O_2_/WR (p < 0.001; r = −0.735), suggesting that the worse the airway obstruction and LVEF, the worse the behavior of these variables in CPET, thus compromising the performance of these HF-COPD patients.

### Cut-off points for FEV_1_ < 1.6 and LVEF < 39%

From this point of the study, our results will be divided by the mean found in our patients FEV_1_ < 1.6 L [n = 21 (52%)] and LVEF < 39% [19 (n = 48%)], our aim in performing this stratification is to demonstrate the impact of diseases on CPET variables and may assist in clinical practice.

The cut-off points, the areas under the ROC curve and 95% CI, as well as the sensitivity and specificity of the clinical variables are shown in Table 3. According to the prediction, the best models of sensitivity and specificity are: OUES < 1.3 and CP < 2383 mmHg.mlO_2_.min^−1^ identified as cut-off points for HF- COPD patients with FEV_1_ < 1.6 L.

**Table 3:** Cut-off values, sensitivity and specificity of hemodynamic response in CPET in HF-COPD with FEV_1_ < 1.6 L.

HF-COPD (N = 21)						
Variables	Cut-off	Sensitivity	Specificity	AUC [CI 95%]	Positive likelihood	Negative likelihood
CP (mmHg.mlO_2_.kg^−1^.min^−1^)	2383	70	53	0.700 [0.501–0.810]	1.39	0.60
OUES	1.3	66	61	0.682 [0.487–0.823]	1.74	0.53

HF: heart failure; COPD: chronic obstructive pulmonary disease; FEV1: forced expiratory volume in the 1 second; CP: Circulatory power and OUES: Oxygen uptake efficiency slope.

**Figure 1:**
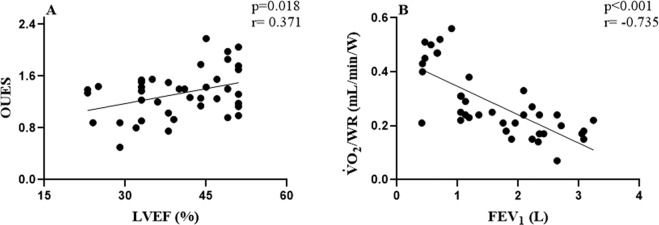
Correlations between pulmonary and cardiac function with metabolic and cardiorespiratory variables in CPET in HF-COPD patients. (**A**) Left ventricular ejection fraction (LVEF) vs OUES; (**B**) Forced expiratory volume in the 1 second (FEV_1_) vs *V̇*O_2_/WR. Pearson correlation test (p < 0.05), N = 40.

The cut-off points, the areas under the ROC curve and 95% CI, as well as the sensitivity and specificity of the clinical variables are shown in Table 4. According to the prediction, the best models of sensitivity and specificity are: OUES: < 1.3, CP < 2116 mmHg.mlO_2_.kg^−1^.min^−1^ and *V̇*_E_/*V̇*CO_2_ slope > 38 identified as cut-off points for HF-COPD patients with LVEF < 39% (Fig. 1).

**Table 4:** Cut-off values, sensitivity and specificity of hemodynamic response in CPET in HF-COPD with LVEF < 39%.

HF-COPD (N = 19)						
Variables	Cut-off	Sensitivity	Specificity	AUC [CI 95%]	Positive likelihood	Negative likelihood
OUES	1.3	70	50	0.701 [0.523–0.862]	1.30	0.66
V̇E/V̇CO_2_ slope	38	71	53	0.610 [0.520–0.788]	1.30	0.66
CP (ml.kg^−1^.min^−1^/W)	2116	70	60	0.762 [0.522–0.803]	1.59	0.56

HF: heart failure; COPD: chronic obstructive pulmonary disease; V̇E/V̇CO_2_ slope: Linear relation between minute ventilation and carbon dioxide production; CP: Circulatory power and OUES: Oxygen uptake efficiency slope.

### Hospitalization analysis

**Figure 2:**
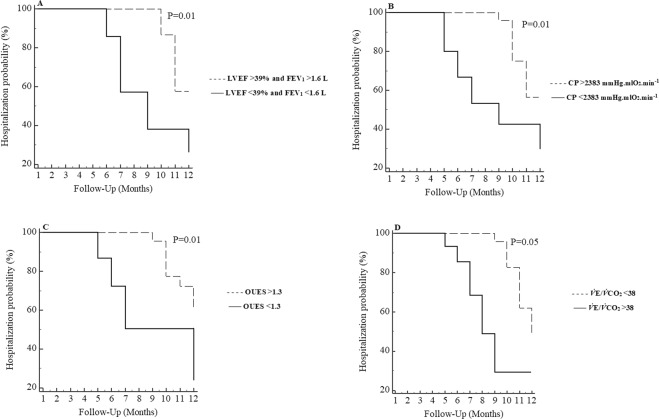
Kaplan-Meier curve for hospitalization according to presence of LVEF < 39% and FEV_1_ < 1.6, CP < 2383 mmHg.mlO_2_.min^−1^, OUES < 1.3 and *V̇*E/*V̇*CO_2_ > 38. LVEF: Left ventricular ejection fraction; FEV_1_: forced expiratory volume in the 1 second. (**A**) LVEF < 39% and FEV_1_ < 1.6 n = 14 and LVEF > 39% and FEV_1_ > 1.6 n = 15; (**B**) CP < 2383 n = 25 and CP > 2383 n = 15; (**C**) OUES < 1.3 n = 22 and OUES > 1.3 n = 18; (**D**) *V̇*E/*V̇*CO_2_ > 38 n = 17 and *V̇*E/*V̇*CO_2_ < 38 n = 23.

In the Kaplan-Meier analysis, when we associated patients with LVEF < 39% and FEV_1_ < 1.6 [n = 14] with a higher probability of hospitalizations over a 12-month period. The hospitalization curve differed significantly in the log-rank test (p = 0.018). In the analysis for the CPET variables, we found that only *V̇*_E_/*V̇*CO_2_ > 38, OUES < 1.3 and CP < 2383 mmHg.mlO_2_.min^−1^ were prognostic indicators for hospitalization (P ≤ 0.05) in COPD-HF patients (Fig. 2).

## Discussion

This is the first study to identify relative cut-off scores to determine prognostic markers of cardiorespiratory worsening and hospitalization in patients through variables of CPET based on COPD and HF severity. The main findings of the present study were: I) Correlations suggest that worsening airway obstruction and LVEF, directly impact in aerobic function (*V̇*O_2_/WR) and ventilatory equivalent oxygen (OUES); II) OUES < 1.3 and CP < 2383 mmHg.mlO_2_.kg^−1^.min^−1^ were identified as cut-off points for HF-COPD patients with FEV_1_ < 1.6 L; III) OUES < 1.3, CP < 2116 mmHg.mlO_2_.min^−1^ and *V̇*_E_/*V̇*CO_2_ slope > 38 were identified as cut-off points for HF-COPD patients with LVEF < 39%; IV) In Kaplan-Meier analysis: LVEF < 39% and FEV_1_ < 1.6, *V̇*_E_/*V̇*CO_2_> 38, OUES < 1.3 and CP < 2383 mmHg.mlO_2_.min^−1^ were prognostic indicators for hospitalization in COPD-HF patients.

We found that airway obstruction and LVEF directly influence aerobic function and OUES. Our study corroborates with Hansen *et al*.^24^, who found a lower *V̇*O_2_/WR in cardiorespiratory disease and suggesting that cardiorespiratory limitation may decrease the load ratio, even with optimal exercise duration.

The main result of our study was to find predictive CPET values for worsening of cardiorespiratory capacity based on airway obstruction in patients with COPD and LVEF in patients with HF and prognostic indicators for hospitalization. We can highlight that through these cutoff points it will be possible to determine diagnosis, training prescriptions, as well as the general state of these patients. De Miguel *et al*.^25^ highlighted that HF-COPD patients have a mixed pulmonary dysfunction. COPD is characterized by airway obstruction and emphysema. In HF, the enlargement of the heart, venous congestion, and interstitial fibrosis compresses the lungs, leading to a restrictive pulmonary disorder^26^. The coexistence of diseases may lead to further increases in disability and mortality, perhaps even impairing the CPET results since variables of the test are poorer in these patients^17–27^.

We emphasize the importance in determining other prognostic indices of cardiorespiratory worsening of CPET in these patients. Another cut-off values in our study were CP < 2383 mmHg.mlO_2_.min^−1^ and CP < 2116 mmHg.mlO_2_.kg^−1^.min^−1^ for FEV_1_ < 1.6 and LVEF < 39% respectively, a new cardiac index recently studied, and an original result for this population. A previous study demonstrated that CP is a surrogate index of cardiac power, shown to have a better prognostic value than peak *V̇*O_2_, *V̇*O_2_/HR and peak SBP in HF^20,22,27^. Physiologically, CP represents the volume of O_2_ added to the mixed venous blood by the lungs and transferred to systemic arterial circulation, against a pressure gradient produced by the heart^22^. CP is related to the central and peripheral components of the cardiac work^16,22^. We emphasize that CP < 2383 mmHg.mlO_2_.min^−1^ was a predictor of hospitalization in these patients.

In COPD, an emphysema burden has been associated with increased *V̇*_E_/*V̇*CO_2_, as a consequence of increased ventilatory drive and greater neuromechanical dissociation^28,29^. In HF, disease progression is associated with higher *V̇*_E_/*V̇*CO_2_ because of an increased ventilatory drive leading to hypocapnia in highly variable combinations^30,31^. Aposto *et al*.^20^, found that increased *V̇*_E_ of the linear *V̇*_E_/*V̇*CO_2_ relationship during ramp-incremental exercise should raise the suspicion of coexistent COPD in patients with HF. The new result of our study is that the *V̇*_E_/*V̇*CO_2_ slope > 38 for patients with LVEF < 39% that was identified as cut-off points and predictor of hospitalization for HF-COPD patients.

Lin *et al*.^32^ found that HF patients with OUES < 1.3 had a higher risk of cardiac events. OUES is considered a method of evaluating cardiopulmonary endurance and it is easily determined when using the breath-by-breath respiratory analysis method^32^. Compared with the values already described in the literature, our result of OUES < 1.3 for patients with FEV_1_ < 1.6 and LVEF < 39%, emphasizes that our study is the first to find a cut-off value for a HF-COPD population.

Thus, our results highlight the importance of identifying predictive values of disease severity-based exercise testing (COPD and HF), as well as the prognosis value of cardiopulmonary exercise testing to help healthcare professionals to identify potential patients for hospitalization. It is possible with cut-off values to obtain a response about the patient’s general health, as we know that hospitalizations limit or even preclude rehabilitation and may aggravate the exercise intolerance of this population.

## Strength and Limitation of the Study

The main limitation in our study is the small number of COPD-HF coexistence, however, despite the high prevalence of COPD and HF, such coexistence has been poorly studied, especially considering the impact of disease severity on exercise capacity. In this sense, future studies should be conducted with a larger sample to confirm our findings^33^.

The strengths of our study include the novelty of finding cut-offs for new rates (CP, OUES and *V̇*_E_/*V̇*CO_2_ slope) and predictors of hospitalization for HF-COPD patients with FEV_1_ < 1.6 L and LVEF 39%. Thus, this is the first study to investigate these indices in HF-COPD patients based on the severity of the diseases. We emphasize as a limitation the sample size, however it is known the difficulty in screening COPD-HF coexistence and that so far no follow-up studies and prognosis of hospitalizations in this population have been conducted, thus highlighting the importance of this work.

## Conclusion

In conclusion, heart failure with reduced left ventricular ejection fraction as well as the severity of COPD impacted negatively selected physiological responses obtained by CPET. CP, OUES and *V̇*_E_/*V̇*CO_2_ slope provided to be useful in practice to clinical interpretation of cardiopulmonary responses during exercise and are a strong risk predictor for hospitalization in HF-COPD coexistence patients.
