# Publisher Correction: The Value of Cardiopulmonary Exercise Testing in Determining Severity in Patients with both Systolic Heart Failure and COPD

**DOI:** 10.1038/s41598-020-64446-x

**Published:** 2020-05-04

**Authors:** Cássia da Luz Goulart, Polliana Batista dos Santos, Flávia Rossi Caruso, Guilherme Peixoto Tinoco Arêas, Renan Shida Marinho, Patricia de Faria Camargo, Tiago da Silva Alexandre, Claudio R. Oliveira, Andréa Lúcia Gonçalves da Silva, Renata Gonçalves Mendes, Meliza Goi Roscani, Audrey Borghi-Silva

**Affiliations:** 1 Cardiopulmonary Physiotherapy Laboratory, Physiotherapy Department, Federal University of Sao Carlos, UFSCar, Rod Washington Luis, KM 235, Monjolinho, CEP: 13565-905, Sao Carlos, SP Brazil; 2 Department of Gerontology, Federal University of São Carlos, São Carlos, Brazil; 3 Department of Physical Education and Health, University of Santa Cruz of Sul, Rio Grande do Sul, Brazil; 4 Department of Medicine, Federal University of São Carlos, SP, Brazil

Correction to: *Scientific Reports* 10.1038/s41598-020-61199-5, published online 09 March 2020

The original version of this Article contained an error in the order of author names, which were incorrectly given as ‘Cássia da Luz Goulart, Polliana Batista dos Santos, Flávia Rossi Caruso, Guilherme Peixoto Tinoco Arêas, Renan Shida Marinho, Patricia de Faria Camargo, Tiago da Silva Alexandre, Claudio R. Oliveira, Andréa Lúcia Gonçalves da Silva, Audrey Borghi-Silva, Renata Gonçalves Mendes & Meliza Goi Roscani’.

Additionally, Meliza Goi Roscani was incorrectly affiliated with ‘Cardiopulmonary Physiotherapy Laboratory, Physiotherapy Department, Federal University of Sao Carlos, UFSCar, Rod Washington Luis, KM 235, Monjolinho, CEP: 13565-905, Sao Carlos, SP, Brazil’. The correct affiliation is listed below.

‘Department of Medicine, Federal University of São Carlos, SP, Brazil’

These errors have now been corrected in the PDF and HTML versions of the Article.

